# Emerging Role of Biologic Drugs Targeting IL-17 and IL-23: Pityriasis Rubra Pilaris

**DOI:** 10.3390/life14080923

**Published:** 2024-07-24

**Authors:** Luca Potestio, Michela D’Agostino, Antonio Portarapillo, Valeria Esposito, Nello Tommasino, Antonia Salsano, Luigi Guerriero, Fabrizio Martora, Matteo Megna

**Affiliations:** Section of Dermatology—Department of Clinical Medicine and Surgery, University of Naples Federico II, 80131 Napoli, Italy

**Keywords:** pityriasis rubra pilaris, biologics, anti-IL17, anti-IL23

## Abstract

Pityriasis rubra pilaris (PRP) is a rare, papulosquamous, inflammatory skin disease. PRP represents a therapeutic challenge. The rarity of this disease and its possible spontaneous remission makes the conduction and interpretation of therapeutic studies particularly difficult. Moreover, PRP not infrequently proves resistant to common topical and conventional systemic therapies. In this context, numerous biologic agents have been reported in PRP treatment. The aim of our manuscript was to review the current literature to evaluate the possible role of biologics targeting the IL17/23 axis in PRP management. Recent cases in the literature have highlighted the use of several promising drugs: IL-17 inhibitors, IL-23 inhibitors, and the IL-12/23p40 inhibitor ustekinumab. However, it should be noted that all these drugs are approved for moderate-to-severe plaque psoriasis and their use in PRP is off label. The treatment of PRP is based on clinical experience, case reports or case series reported in the literature, as randomized controlled trials are difficult to conduct due to the rarity of the condition. Despite data on the efficacy of drugs targeting IL-17 and IL-23 being promising, they are still limited. Certainly, further studies are desirable to better characterize PRP and establish shared guidelines.

## 1. Introduction

Pityriasis rubra pilaris (PRP) is a rare, papulosquamous, inflammatory skin disease [1]. It is usually characterized by follicular hyperkeratotic papules converging into widespread orange–red scaly plaques, in association with typical islands of unaffected skin and palmoplantar keratoderma [1,2,3]. This idiopathic disease can afflict both adult and pediatric populations, with the first main peak in childhood (between the ages of 1 and 10 years) and the second one in adulthood (between the ages of 50 and 60 years) [3]. There is a lack of precise data regarding the incidence of PRP, which is estimated to be between 1:5000 and 1:50,000 per year [1,4,5]. The etiopathogenesis is still unclear, even if several predisposing factors have been proposed, including genetic factors, abnormalities in vitamin A metabolism, dysregulated immune response to superantigens, infections, and other triggers such as drug exposure, malignancies, and autoimmune diseases [1,2,3,4,6]. Familial cases of PRP are associated with mutation of the CARD14 gene, located in the psoriasis susceptibility locus 2 (PSORS2), which codifies a protein that activates the nuclear factor (NF)-кB, responsible for enabling genes involved in immune and inflammatory reactions [7,8,9,10]. Other genes that have been suggested to be associated with PRP were DTX1, encoding a ubiquitin ligase that sustains T-regulatory cells; NLRC5, responsible for the transcription of major histocompatibility I complex (MHC-I); and ABCA12, responsible for the transport of lipids across the keratinocyte plasma membrane [7,8,9,10]. However, further studies are needed to confirm these data. Immune dysregulation has also been investigated in PRP pathogenesis. In particular, the role of the IL23/17 axis seems to be the major culprit, as well as it is well known that CARD14 mutation enhances this axis. Moreover, both innate and adaptative inflammatory pathways seem to be more activated in patients with PRP compared with healthy controls [7,8,9,10,11].

As regards the role of vitamin A, it was previously believed that its deficiency could be correlated with PRP, but recently it has been suggested that it is instead an altered metabolism that is predisposed to the development of PRP, as the serum levels of vitamin A and those of its carrier, the retinol-binding protein, are frequently normal in PRP patients [1,2,3,12]. In addition, PRP can occur in concomitance with autoimmune diseases such as vitiligo, celiac disease, myasthenia gravis, and autoimmune thyroiditis [8,9,10,11,12,13,14,15,16], and can be associated with infections, including those such as HIV, SARS-CoV-2 virus, EBV, VZV, and hepatitis A virus [17,18,19,20,21,22]. Iatrogenic factors that can trigger PRP include medications [23,24,25,26,27] and vaccines [28,29,30,31,32,33,34]. Several cases of PRP are reported in association with malignancies, even as an initial manifestation [35,36,37,38,39]. Griffiths proposed the classification of PRP into five types based on clinical features, age of onset, and prognosis: classic adult (type I), atypical adult (type II), classic juvenile (type III), circumscribed juvenile (type IV), and atypical juvenile (type V) [5]. Later, a type VI of PRP was recognized in association with HIV infection [17]. The main differential diagnoses are psoriasis, generalized hypersensitivity reaction, cutaneous T-cell lymphoma, subacute cutaneous lupus erythematosus, follicular eczema, lichen planopilaris, follicular ichthyosis, seborrheic dermatitis and atopic eczema [1,2]. The diagnosis of PRP is based on the finding of typical clinical and histopathological features [1], which include alternating orthokeratosis and parakeratosis vertically and horizontally (checkerboard pattern), focal or confluent hyper granulosis, thick suprapapillary plates, sparse superficial perivascular infiltrates, broad rete ridges and follicular plugs [1,40,41]. Recently, the diagnostic aid of non-invasive methods such as confocal reflectance microscopy has also been evaluated, revealing a good concordance rate with histopathological examination [42,43]. Treatment is based on clinical experience and case reports or case series reported in the literature, as randomized controlled trials are difficult to conduct due to the rarity of the condition [3,43]. Furthermore, although great progress has been made in treating the most common inflammatory skin diseases, such as psoriasis and atopic eczema, the same has not been achieved for rare inflammatory cutaneous diseases [3]. The aim of our study is to evaluate the emerging role of biological therapies in the treatment of PRP, with a particular focus on drugs targeting IL-17 and IL-23.

## 2. Current Therapeutic Panorama

PRP represents a therapeutic challenge. The rarity of this disease and its possible spontaneous remission makes the conduction and interpretation of therapeutic studies particularly difficult [1]. Moreover, PRP not infrequently proves resistant to common topical and conventional systemic therapies [44]. The choice of treatment is generally based on the extension and severity of the pathology [43].

### 2.1. Topical Treatments

Topical treatments include corticosteroids, emollients, keratolytics, calcipotriol, and topical tretinoin [3,44] and are used in localized forms or in combination with systemic therapies in severe forms to alleviate symptoms [43,44]. Topical tazarotene has also been considered a valid therapy [45]. Furthermore, pimecrolimus cream 1% has proven to be a viable alternative to corticosteroids [46] and topical capsaicin solution 0.03% in the treatment of itching, where antihistamines have been unsuccessful [47]. The most commonly used keratolytics include 5–10% urea and 5% salicylic acid and must be considered in the presence of hyperkeratosis and palmoplantar keratoderma [1,43].

### 2.2. Systemic Treatments

As regards systemic therapies, a recent study by Ross et al. showed that the most widely used are systemic retinoids, methotrexate, and phototherapy, respectively [48]. Systemic retinoids are currently considered the first-line therapy in the treatment of PRP, both in adult and juvenile forms [3,43]. The most widely used are isotretinoin and acitretin [1,3,43], although positive results have also been reported with alitretinoin [49,50], even in the treatment of juvenile forms of PRP [51,52,53]. Retinoid clinical response is usually evident after 3–6 months of therapy, but in certain cases, longer treatment is necessary [43]. In clinical practice, isotretinoin is used at a dosage of 0.5–1 mg/kg per day [3,43], while acitretin is at a dosage of 0.5–0.75 mg/kg per day [43]. High-dose retinoid therapy is frequently not well tolerated due to its side effects, mainly mucocutaneous ones [43]. Moreover, the use of oral retinoids may be related to possible adverse events such as alterations in the lipid profile, and it should be kept in mind that retinoids are teratogenic and pregnancy should be avoided until three years after their discontinuation [42,43]. Retinoids can also be successfully associated with phototherapy, as reported in some cases in the literature, including between acitretin and PUVA, NBUVB, or UVA1 [1,3]. Generally, both UVB and PUVA therapy are commonly used treatments for PRP [1,43], although the results are very variable [48]. The use of phototherapy can be evaluated in widespread disease if other systemic therapies are contraindicated or refused by the patient. Because of the possible occurrence of photoaggravated PRP, the use of pre-treatment phototesting should be considered [1,3]. In case of inadequate response to retinoids, methotrexate (MTX) has long been proposed as a second line of therapy, at a dosage from 15 to 25 mg per week, similar to that suggested for psoriasis, with individual adjustment according to the patient features [3,54]. A review by Koch et al. has shown good response rates and safety of MTX in the treatment of PRP, by acting on the reduction of proinflammatory cytokines and depression of the mitotic rate of keratinocytes [54]. However, MTX is not always successful for PRP or clinically sufficient in monotherapy, as reported in several cases in the literature [49,55,56]. Furthermore, the use of MTX may be related to possible adverse events such as myelosuppression, hepatotoxicity, nephrotoxicity, etc., and it should be kept in mind that MTX is teratogenic [55,56].

Other immunosuppressive agents, such as azathioprine and cyclosporine, have been effective in some cases of PRP [1,43], but their use has not always proved sufficient. Other miscellaneous therapies reported in the literature but limited to a small number of patients or individual case reports are mycophenolate, penicillin, fumarates, extracorporeal photopheresis, and intravenous immunoglobulin [1,3].

### 2.3. Biologic Agents and Small Molecules

In the management of PRP refractory cases with conventional systemic therapies, the introduction of biologic drugs has represented an effective second-line treatment [4,57], and also a promising first-line therapy [1,58]. However, multicenter randomized clinical trials are missing and their use is off label [43]. Biologic agents that have been successfully administered include anti-tumor necrosis factor (TNF)-alpha drugs and, recently, inhibitors of the interleukin (IL)-23/IL-17 pathway [3,4,43]. It should be noted that, to date, these drug classes have been in the treatment of psoriasis, which shares clinical and histopathological similarities to PRP [3]. Anti-TNF-alpha agents used in PRP treatment include infliximab, etanercept, and adalimumab [1,3,4], and, over the past 10 years, have proven effective in treating both adult and juvenile forms of PRP [43]. Of note, data on the use of certolizumab in PRP are absent. A review by Petrof et al. illustrated the excellent response rate to treatment with anti-TNF-alpha, reporting that out of a total of 15 cases, there was a complete response in 12 patients, a partial response in 2, and a response was absent in 1 [59]. Six patients received the biologic agent as exclusive therapy, while nine received the drug in combination with MTX or acitretin [59]. In a retrospective analysis, the mean interval between initiation of TNF inhibition and notable clinical response is 5.7 weeks [60]. A review by Napolitano et al. reveals that, among anti-TNF-alpha agents, the most widely used is infliximab at the prevalent dose of 5 mg/kg, for a variable number of weeks, while the second one is etanercept [4]. A paradoxical reaction following the administration of infliximab therapy for Takayasu’s arteritis with the development of PRP-like eruption has been reported in the literature [61]. Furthermore, it is interesting to note that a failure of treatment with a single anti-TNF-alpha does not preclude a secondary response to other agents of the same class [1]. Of interest, cases of refractory PRP treated with apremilast, an oral, small-molecule PDE4 inhibitor approved for the treatment of moderate-to-severe plaque psoriasis and psoriatic arthritis, have been reported in the literature [62,63].

Recently, there has been growing interest in the use of inhibitors of the IL-23/IL-17 pathway for PRP treatment [3,4,43,64]. A report by Feldmeyer et al. identifies the role of the IL-23–TH17 axis in PRP, highlighting upregulated expression for most proinflammatory innate cytokines, such as TNF, IL-6, IL-12, IL-23, IL-1β, and, among adaptive T-cell cytokines, an increase in T-Helper (TH)-1 cytokines and TH17 cytokines IL-17A, IL-17F, and IL-22, in lesional PRP skin samples [65]. These results emphasize the rationale for targeting the IL-23–TH17 pathway as a treatment option for refractory PRP [3,65]. In this context, numerous biologic agents have been reported in PRP treatment and we aim to analyze them. Recent cases in the literature have highlighted the use of several promising drugs: IL-17 inhibitors, including secukinumab, ixekizumab, brodalumab, and bimekizumab; IL-23 inhibitors, such as risankizumab, guselkumab, and tildrakizumab; and the IL-12/23p40 inhibitor ustekinumab [66,67].

## 3. Material and Methods

A review of the current literature was carried out by searching the following keywords on the main databases (Embase, PubMed, Cochrane Skin, and clinicaltrials.gov) until 30 May 2024: “pityriasis rubra pilaris”, “treatment”, “biologic”, “secukinumab”, “ixekizumab”, “brodalumab”, “bimekizumab”, “ustekinumab”, “guselkumab”, “risankizumab”, and “tildrakizumab”.

All article types (reviews, meta-analyses, clinical trials, real-life studies, case series, and case reports) were screened. A revision of the bibliography was also performed to include articles that could have been missed. Articles regarding other treatments for PRP were excluded. The current article is based on previously conducted studies and does not contain any studies with human participants or animals performed by any of the authors.

## 4. Results

The results are summarized in Table 1.

### 4.1. Secukinumab

Secukinumab is a human immunoglobulin G1 monoclonal antibody against IL-17A. Boudreaux et al. conducted an open-label, single-arm clinical trial in twelve patients with PRP who received a 24-week course of secukinumab. The clinical trial showed great clinical efficacy. Indeed, at week 28, 6 of 11 patients (55%) achieved Psoriasis Area Global Assessment (PASI) 75, and 3 patients (27%) achieved PASI 90. Similarly, PGA (*p* = 0.008) and Dermatology Life Quality Index (DLQI) scores (*p* = 0.010) showed significant improvement with treatment. No serious side adverse events were encountered [68].

Abduljawad et al. reported the treatment of 49 patients with PRP with secukinumab, which showed an almost 90% complete improvement among all types of PRP [64]. The dose administered was 300 mg weekly for five weeks, then monthly, and the average time of therapy was six months [64]. Secukinumab showed a more significant response in types I, III, and IV: 6 patients with type III and IV had a 100% complete clinical response, and 21/23 patients with type 1 experienced complete remission of disease [64]. Only 57% of patients with type 2 PRP obtained a clinical response to secukinumab; 13 patients with an unknown type of PRP had 100% remission [64]. Several case reports in the literature show the efficacy of secukinumab in the treatment of PRP, as described by Liang et al., who reviewed 9 studies including 12 adult patients with PRP (9 cases of type I, 2 cases of type II, and 1 case of type III) treated with secukinumab [69]. Among them, 11 cases all failed to respond sufficiently to at least one line of previous therapy, such as topical corticosteroids (steroids), NB-UVB, MTX, isotretinoin, acitretin, cyclosporine, infliximab, ustekinumab, and adalimumab [69]. A fully or almost complete response has been shown in nine patients (75%) and an excellent response in two patients (16.7%), with an overall clinical response rate of 91.7% (11/12) [69]. There were no side adverse effects or relapse during and after treatment [69].

### 4.2. Ixekizumab

Ixekizumab is a humanized monoclonal IgG4 antibody that binds IL-17A. In a single-armed trial, Haynes et al. proved that ixekizumab reduced clinical signs and symptoms of PRP in a subset of patients, including those unresponsive to other systemic therapies. A total of 12 patients were enrolled, with 11 completing the full course of therapy. It is of interest that 11 of 12 patients (92%) were refractory to previous therapy; 3 of 12 (25%) participants had 1 previous systemic therapy fail; 2 of 12 (17%) had 2 previous systemic therapies fail; and 6 of 12 (50%) had 3 or more previous systemic therapies fail. Seven of twelve patients (58%) achieved PASI50 from baseline at week 24 and no serious or unexpected adverse events. The mean (SEM) improvement in affected body surface area from baseline to week 24 was 29.8% (9.3%). The four participants who had the most improvement in PASI score at week 24, ranging from 89% to 100% improvement, remained in remission of therapy at week 36 [70].

Abduljawad M et al. reported the use of anti-IL-17A for the treatment of PRP. The population treated with Ixekizumab included 28 patients, of which 8 patients with PRP type I, 5 patients with PRP type II, 1 patient with PRP type III, 1 patient with PRP type IV, and 13 patients with PRP of an unknown type. Ixekizumab was administered subcutaneously at a dosage of 160 mg once, followed by 80 mg every 2 weeks for 12 weeks and then 80 mg every 4 weeks. The results show that 15 patients with PRP type I, II, III, and IV had a complete response to treatment, while in patients with unknown PRP type, there was a complete response in only 18% of patients (n: 2), and, in 82% (n: 11), the response was incomplete or absent [70]. There are several case reports in the literature describing the efficacy of ixekizumab in the treatment of pityriasis rubra pilaris [71,72,73,91,92].

Kranyak A. et al. described a case report of a 53-year-old HIV+ male patient affected by PRP unresponsive to several courses of systemic corticosteroids treated with ixekizumab (160 mg once followed by 80 mg every two weeks). After the first week of treatment, a clinical partial response was achieved and, at week 4, a complete clearance of lesions was achieved [71]. A few articles report the efficacy of ixekizumab in the treatment of PRP occurring after vaccination for COVID-19. Liu YT et al. reported a case report of a 67-year-old man with PRP occurring 7 days after vaccination for COVID-19 unresponsive to systemic retinoids (acitretin) treated with ixekizumab (160 mg once, followed by 80 mg every 2 weeks). At week 4, a partial clinical response was obtained. At week 12, a complete clinical response was obtained [72].

Zhao P et al. described a case report of a 54-year-old female affected by PRP that occurred 2 weeks after the Pfizer-BioNTech COVID-19 booster vaccine. She was previously treated with topical and systemic corticosteroids and with dupilumab without clinical benefit. Subsequently, she was treated with ixekizumab (160 mg once then 80 mg every two weeks), achieving a complete clinical clearance after 2 months of treatment [73]. 

### 4.3. Brodalumab

Brodalumab is a human monoclonal antibody that targets the IL-17 receptor A subunit (IL-17RA) and blocks signaling of not only IL-17A but also IL-17F, IL-17C, IL-17E, IL- 17F, and IL17-A/F. There are few clinical cases in the literature proving the use of brodalumab in PRP. De Felice et al. described the efficacy of brodalumab in a 52-year-old female with familial PRP subtype (type V of Griffith classification), previously treated with etanercept and ustekinumab, interrupted due to ineffectiveness. The skin disease significantly improved after 1 month of treatment (210 mg s.c. every 2 weeks after three loading doses at weeks 0, 1, and 2) with a complete response at week 8 and no relapse. No side effects were experienced throughout the course of therapy [74]. Also, Amat-Samaranch et al. reported the response of type 1 PRP to brodalumab after primary failure to ciclosporin, acitretin, and ustekinumab in a 36-year-old man. The efficacy and rapid onset of action of brodalumab were promptly noted, with complete response after 10 weeks of treatment and satisfactory relief of pruritus at 4 weeks [75].

Another case report demonstrated that brodalumab is effective in the treatment of therapy-resistant pityriasis rubra pilaris. A 60-year-old man affected by PRP unresponsive to cyclosporin was treated with brodalumab 210 mg by subcutaneous injection with a complete clinical response obtained after 8 weeks (BSA 0) and maintained with a 6-month follow-up [76].

### 4.4. Bimekizumab

Bimekizumab is a novel humanized monoclonal IgG1 antibody inhibiting IL-17A and IL-17F. It was recently approved for the treatment of moderate-to-severe plaque psoriasis in adults [93,94,95].

The recent approval of bimekizumab means that there is limited literature about its off-label use for PRP treatment. Saad M et al. reported a case report of a 42-year-old patient with PRP that occurred after the third dose of the SARS-CoV-2 vaccine. Previously, he was treated with several courses of systemic and topical steroids and with cyclosporine for 6 weeks without any benefit. Following therapeutic failures, bimekizumab was administered at a dosage of 320 mg every 4 weeks for five injections and then every 8 weeks. At week 32, a complete clinical response was achieved [77].

### 4.5. Ustekinumab

Ustekinumab is a human monoclonal antibody targeting the p40 subunit that inhibits interleukins IL-12 and IL-23 involved in the pathogenesis of several diseases including psoriasis and PRP [78].

Rawal S. et al. describe the off-label use of ustekinumab in the treatment of various diseases. They reported the efficacy of ustekinumab in the treatment of PRP in patients with a CARD14 mutation. In the first study conducted by Craiglow et al., five patients were treated with ustekinumab, showing that in four of the five patients (80%) a complete response was achieved, while in one patient (20%), a partial response was obtained. Another study by Lwin et al. described the treatment of two patients with a CARD4 mutation with ustekinumab. At week 12, one patient showed an improvement in PASI and DLQI from 25.7 to 100 and from 18 to 0, respectively, and the other patient showed a decrease in PASI and DLQI from 29.2 to 48 and from 22 to 3, respectively [96]. Napolitano et al. reported the efficacy and safety of ustekinumab in the treatment of 18 PRP patients. Ustekinumab was administered at a dosage of 45 mg every 4 weeks for 4 weeks, and then every 8 weeks. The population included 12 patients with PRP type I, 1 patient with PRP type II, 1 patient with PRP type III, 3 patients with familial PRP, and 1 patient with an unknown PRP type. A complete clinical response was observed in 78% of patients (n: 14), a partial response was observed in 11% of patients (n: 2) and no clinical benefit was observed in 11% of patients (n: 2). No side effects were observed in the whole population and only one relapse was observed [4]. Several case reports concerning the efficacy of ustekinumab have been described. Vieira Granja B et al. described a case of a 69-year-old female affected by PRP that occurred 2 days after the first dose of the Pfizer-BioNTech COVID-19 vaccine. The patient was previously treated with methotrexate, methotrexate combined with PUVA therapy, and retinoids (acitretin), without any benefit and with an increase in transaminases. Ustekinumab was administered at a dosage of 45 mg at week 0, week 4, and then every 12 weeks. After the first two injections (week 4), a complete clinical response was achieved and still provided excellent disease control for several months [82]. Di Stefani et al. discussed a case report of a 31-year-old male affected by PRP treated with ustekinumab. Previous systemic treatments including cyclosporine, methotrexate, and acitretin failed. After the histological diagnosis of PRP type 1, ustekinumab was administered at the dosage of 45 mg every 4 weeks for 4 weeks and then every 8 weeks. Clearance of disease was achieved at week 8 and it retained the control of disease until week 64 [83]. Several case reports confirmed the efficacy and safety of ustekinumab in the treatment of PRP candidate ustekinumab, among biologics, as the most possible therapeutic alternative for PRP.

### 4.6. Guselkumab

Guselkumab is a fully human immunoglobulin G1 λ monoclonal antibody that selectively binds and inhibits the p19 subunit of IL-23. A single-arm, investigator-initiated nonrandomized trial evaluated the efficacy and safety of Guselkumab for the treatment of moderate-to-severe PRP, enrolling 14 adults with moderate-to-severe PRP. Of these, only 12 completed the study. The primary outcome was observed at week 24. The mean improvement in PASI score, pruritus, and DLQI score was 61.8% (*p* < 0.001), 62.3% (*p* = 0.001), and 60.2% (*p* < 0.001), respectively. Nine participants (75%) achieved a 50% improvement in PASI. Among these clinical responders, at week 36, 8 of 9 achieved PASI75, and 6 of 9 achieved PASI90. No participants had pathogenic CARD14 gene variations. There was one serious adverse event that was not associated with the study drug [78]. Although limited existing trials, there are several case reports reporting the efficacy of guselkumab in the treatment of PRP [79,80,84].

Pilz AC et al. reported a case of two patients, a 65-year-old male and a 75-year-old male with PRP involving 95% and 98% of the skin surface, respectively. Following histological diagnosis guselkumab was administered subcutaneously at a dosage of 100 mg every 2 weeks for 4 weeks and then 100 mg every 8 weeks. The patients had almost complete clearance of the lesions at week 16, DLQI decreased from 15 to 1 (patient 1) and from 19 to 2 (patient 2), and the static physician global assessment decreased from 5 to 1 [84].

Nishimura M et al. reported a case report of a 60-year-old male affected by PRP. The patient was previously treated with oral methotrexate and retinoids, without any benefit, and then brodalumab was administered with a worsening of the disease. The multi-drug failure diagnosis was histologically confirmed. Oral cyclosporine (100 mg/day) was administered with a partial clinical benefit and then it was combined with guselkumab subcutaneously at the dosage of 100 mg every 4 weeks and then 100 mg every 8 weeks. At week 12, a complete clearance of lesions was achieved. The clinical response was retained for more than 1 year even after cyclosporin interruption [79].

Nagai H et al. reported a case report of a 47-year-old female affected by PRP of the face, trunk, arms, and palms. Several treatments were administered, including oral etretinate (30 mg/day), which was stopped due to liver injury, and oral apremilast combined with bath-PUVA and topical steroids, without any benefit. Subsequently, guselkumab (100 mg every 4 weeks for 4 weeks followed by 100 mg every 8 weeks) was administered. At week 24, a complete clinical response was achieved with a decrease in BSA from 90% to 2% and sPGA from 5 to 1. After the clearance of skin lesions subsequent injections were discontinued without relapse after 36 weeks from the last one administered [81].

The studies in the literature report a good efficacy and safety of guselkumab in the treatment of PRP and it may in the future represent a valid therapeutic alternative for non-responsive forms.

### 4.7. Risankizumab

Risankizumab is a humanized monoclonal antibody that selectively inhibits interleukin (IL)-23 binding IL-23p19 subunit [97]. Ricar et al. reported successful treatment of extensive PRP type I with risankizumab in a 51-year-old woman with a baseline BSA of 75% and DLQI of 26. After 12 weeks of treatment with Risankizumab (150 mg at weeks 0 and 4 and then every 12 weeks), BSA decreased to 3% and DLQI to 2, until reaching the value, at week 32, of 1% and 0, respectively. At week 52, the clinical findings remained unchanged and risankizumab was still providing excellent disease control without adverse effects [85].

Risankizumab also showed good efficacy and safety among pediatric patients with PRP, as shown by Kaminska et al., who describe the use of risankizumab (75 mg) in three pediatric patients with PRP. Of these, two patients showed almost complete clearance of skin lesions at week 4, while the third patient achieved clearance of lesions after the second administration of risankizumab. No adverse effects were reported [98].

Among the growing number of case reports demonstrating the efficacy of risankizumab for the treatment of both juvenile and adult-onset PRP, it is interesting to highlight the case presented by Koroneos et al. of juvenile-onset PRP in an adult patient successfully treated with singular risankizumab therapy after developing anaplastic large cell lymphoma (ALCL). The 47-year-old male patient was unresponsive to acitretin, methotrexate, cyclosporine, and secukinumab and was diagnosed with ALCL stage 1A years during ixekizumab therapy. Two months after completing chemotherapy, risankizumab was administered at the dose of 150 mg subcutaneously every 8 weeks. A relevant decrease in PASI and DLQI from 28.6 and 15 to 6 and 9 was observed at week 36, respectively [86].

### 4.8. Tildrakizumab

Tildrakizumab is a humanized IgG1 monoclonal antibody that selectively binds the p19 subunit of IL-23 inhibiting its interaction with the receptor [99]. There is little data in the literature regarding the efficacy and safety of tildrakizumab in PRP, but case reports have recently been published showing good clinical response after tildrakizumab treatment [87,88,89,90,100].

Holmes et al. reported 3 cases of treatment-resistant PRP successfully treated with tildrakizumab [87]. Other 2 cases have been reported by Villa-Gonzalez et al. [88]. Moreover, Licata et al. reported the case of a 55-year-old man affected by PRP unresponsive to acitretin and methotrexate who was undertaking treatment with tildrakizumab 100 mg by subcutaneous injection at weeks 0 and 4 and then quarterly. A complete clinical response was obtained after the third injection (BSA 0; PGA 0) and was retained for 6 months [90]. Finally, Zagarella et al. reported another case [89].

## 5. Conclusions

The management of PRP may be challenging. Indeed, the rarity of this condition does not allow the establishment of shared guidelines since consistent data are lacking. Moreover, PRP pathogenesis is still unclear, making it difficult to refer to targeted therapies. Finally, the possible spontaneous remission of PRP makes the conduction and interpretation of therapeutic studies particularly difficult. It is of interest that the use of peripheral flow cytometry may be useful in the diagnostic process [87]. In this context, the introduction of new drugs is mandatory since PRP is not infrequently unresponsive to common topical and conventional systemic therapies.

The recent development of biologic drugs selectively targeting the IL23/17 axis is a promising option. Anti-IL17 and anti-IL23 drugs for moderate-to-severe psoriasis have several real-life data. Moreover, more and more case reports and case series on the use of the most recently approved biologic drugs targeting IL17 and IL23 are enriching the literature. However, it should be noted that all these drugs are approved for moderate-to-severe plaque psoriasis and their use in PRP is off label.

Furthermore, the most used score for assessing the severity of PRP and clinical improvement is derived from psoriasis (PASI), leading to the need for specific tools able to correctly evaluate and characterize the severity of PRP. Finally, more studies will elucidate if the clinical response may also be related to the specific phenotype of PRP as well as to the CARD14 mutation.

To sum up, the treatment of PRP is based on clinical experience, case reports, or case series reported in the literature, as randomized controlled trials are difficult to conduct due to the rarity of the condition. Despite data on the efficacy of drugs targeting IL-17 and IL-23 being promising, they are still limited. Certainly, further studies are desirable to better characterize PRP and establish shared guidelines. 

## Figures and Tables

**Table 1:** Main manuscripts regarding the use of anti-IL17 and anti-IL23 on PRP management.

Authors	Drug	Patients	PRP Type	Dose	Response
Boudreaux et al. [68]	Secukinumab	12	-	-	11 (55%): PASI 75 at week 28 3 (27%): PASI 90 at week 28
Abduljawad et al. [64]	Secukinumab	49	1, 2, 3, 4 type 13 unknown type	300 mg weekly for 5 weeks, then monthly for 6 months	6 type III and IV, 21 type 1 57% of type II, and 13 unknown types had complete response
Liang et al. [69]	Secukinumab	12	9 type I 2 type II 1 type III	-	9 (75%) complete response 2 (16.7%) excellent response
Haynes et al. [70]	Ixekizumab	12	-	-	7 (58%): PASI50 at week 24 with 4 remained in remission off therapy at week 36
Abduljawad et al. [64]	Ixekizumab	28	8 type I 5 type II 1 type III 1 type IV 13 unknown type	160 mg once, then 80 mg every 2 weeks for 12 weeks, and then 80 mg every 4 weeks	15 types I, II, III, and IV complete 2 (18%) unknown types complete response 11 (82%) unknown, 82% (n: 11) incomplete or absent response
Kranyak et al. [71]	Ixekizumab	1	-	160 mg once, then 80 mg every two weeks	Complete response after 4 weeks
Liu et al. [72]	Ixekizumab	1	-	160 mg once, then 80 mg every two weeks	Complete response after 12 weeks
Zhao et al. [73]	Ixekizumab	1	-	160 mg once, then 80 mg every two weeks	Complete response after 2 months
De Felice et al. [74]	Brodalumab	1	Type V	210 mg every 2 weeks after three loading doses at weeks 0, 1, and 2	Complete response at week 8
Amat-Samaranch et al. [75]	Brodalumab	1	Type 1	-	Complete response after 10 weeks
De Rosa et al. [76]	Brodalumab	1	-	210 mg	Complete response after 8 weeks
Saad et al. [77]	Bimekizumab	1	-	320 mg every 4 weeks for 5 injections and then every 8 weeks	Complete response after 32 weeks
Velasco et al. [78]	Guselkumab	14 (only 12 completed the study)	-	24 weeks	9 (75%) achieved a 50% improvement in PASI 8: PASI75 at week 366. PASI90 at week 36
Pilz et al. [79]	Guselkumab	2	-	100 mg every 2 weeks for 4 weeks, then 100 mg every 8 weeks	Complete response at week 16
Nishimura et al. [80]	Guselkumab	1	-	100 mg every 4 weeks and then 100 mg every 8 weeks	Complete clearance of lesions at week 12
Nagai et al. [81]	Guselkumab	1	-	100 mg every 4 weeks and then 100 mg every 8 weeks	Complete clinical response at week 24
Rawal et al. [82]	Ustekinumab	5 with a CARD14 mutation.	-	-	4 (80%) complete response 1 (20%) partial response
Lwin et al. [73]	Ustekinumab	2 with a CARD14 mutation.	-	-	1: PASI from 25.7 to 100 at week 12 1: PASI from 29.2 to 48 at week 12
Napolitano et al. [4]	Ustekinumab	18	12 type I 1 type II 1 type III 3 familial PRP 1 unknown type	45 mg every 4 weeks for 4 weeks, then every 8 weeks	14 (78%): complete clinical response 2 (11%): partial response 2 (11%): no clinical benefit
Vieira Granja et al. [83]	Ustekinumab	1	-	45 mg at week 0, week 4, and then every 12 weeks	Complete clinical response at week 4
Di Stefani et al. [84]	Ustekinumab	1	Type 1	45 mg every 4 weeks for 4 weeks and then every 8 weeks	Complete clinical response at week 8 with control of disease until week 64
Ricar et al. [85]	Risankizumab	1	Type 1	150 mg at weeks 0 and 4 and then every 12 weeks	BSA decreased from 75% to 3% after 12 weeks and to 1% after 32 weeks
Kolt-Kaminska et al. [73]	Risankizumab	3	-	75 mg	2: almost complete response at week 4 1: clearance after the second administration
Koroneos et al. [86]	Risankizumab	1	Juvenil onset	150 mg every 8 weeks.	PASI decreased from 28.6 and 15 at week 36
Holmes et al. [87]	Tildrakizumab	3	-	200 mg at weeks 0, 4, and then quarterly	1: PASI100 at 6 months 1: PASI100 at 4 months 1: PASI100 at 9 months
Villa-Gonzalez et al. [88]	Tildrakizumab	2	-	100 mg at weeks 0, 4, and then quarterly	PASI100 at week 4
Zagarella et al. [89]	Tildrakizumab	1	-	200 mg at weeks 0, 4, and then quarterly	PASI100 at week 8
Licata et al. [90]	Tildrakizumab	1	-	100 mg at weeks 0, 4, and then quarterly	Complete clinical response after the third injection and retained for 6 months

## Data Availability

The data that support the findings of this study are available on request from the corresponding author.
